# Possibilities of Using a Multispectral Camera to Assess the Effects of Biostimulant Application in Soybean Cultivation

**DOI:** 10.3390/s25113464

**Published:** 2025-05-30

**Authors:** Paweł Karpiński, Sławomir Kocira

**Affiliations:** 1 Department of Machine Operation and Production Processes Management, University of Life Sciences in Lublin, 20-612 Lublin, Poland; 2 Faculty of Agriculture and Technology, University of South Bohemia in České Budějovice, 370-05 České Budějovice, Czech Republic

**Keywords:** drone, multispectral camera, vegetation index, natural biostimulator, soybean

## Abstract

Soybean cultivation plays a crucial role in the global food system, providing raw materials for both the food and feed industries. To enhance cultivation efficiency, plant biostimulants are used to improve metabolism and stimulate growth. A key aspect of modern cultivation is the ability to rapidly and non-invasively assess crop status. One such method involves the use of drones equipped with multispectral cameras. This paper presents the results of an experimental study on soybean cultivation involving a natural biostimulant in the form of *Epilobium angustifolium* extract (commonly known as fireweed) and a commercial seaweed-based biostimulant, Kelpak. The research was conducted at an experimental farm in eastern Poland. The effectiveness of the preparations was evaluated using a drone-mounted multispectral camera. Changes in the values of selected spectral indices were analyzed: the Normalized Difference Red Edge Index (NDRE), the Leaf Chlorophyll Index (LCI), and the Optimized Soil-Adjusted Vegetation Index (OSAVI). The study included a control group treated with pure water. Mathematical and statistical analyses of the mean values and standard deviations of the indices were conducted. The results demonstrated that multispectral scanning allows for the detection of significant differences between the effects of the *E. angustifolium* extract, the seaweed-based biostimulant, and the water control. These findings confirm the utility of this method for assessing the effectiveness of biostimulant applications in soybean cultivation.

## 1. Introduction

Soybean cultivation holds a crucial position in global agriculture due to its high nutritional value. As a major source of protein and fats, soybeans are the subject of ongoing research aimed at improving yields under diverse environmental conditions. While primarily used as animal feed, soybeans have gained increasing popularity in the food industry in recent years. The world’s largest soybean producers include Brazil, the United States, Argentina, India, and China, collectively accounting for over 92% of global production [1]. In light of the growing demand for soybean products, the implementation of modern crop monitoring technologies has become essential for achieving high yields and promoting sustainable agricultural practices. This is particularly important given the crop’s susceptibility to stress, which can negatively impact growth and yield [2].

One innovative approach in crop management involves the use of biostimulants, which can positively influence plant development. These substances are known to enhance plant metabolism, stimulate growth, improve resistance to abiotic stress, and increase nutrient uptake. The mechanisms of action of biostimulants include the modulation of plant hormone levels, the activation of enzymes involved in chlorophyll synthesis, and the enhancement of natural plant defense mechanisms. These physiological responses affect the plant’s health status, which can vary throughout the growing season. Research presented in [3] demonstrated that foliar application of a biostimulant can improve soybean yield by 25% without negatively affecting seed nutritional value. Biostimulants can be of synthetic or natural origin. The choice between synthetic and natural biostimulants depends on various factors, including crop type and environmental conditions. The impact of synthetic biostimulants (“Atonik” and “Tytanit”) on the growth, yield, and nutraceutical and antioxidant properties of soybeans is discussed in [4]. The findings indicate that biostimulants can positively influence soybean yield while also modifying nutrient content and antioxidant activity of the seeds. The application of a natural biostimulant based on the seaweed *Ascophyllum nodosum* (L.) Le Jolis showed significant effects on all growth and yield parameters of soybean [5]. Furthermore, the use of natural biostimulants can mitigate the effects of heat stress [6]. The response to heat stress may depend on the application of an amino-acid-based biostimulant containing macro- and microelements [7]. As the research results presented in [8] demonstrate, biostimulants, particularly “Improver,” significantly influence the yield and yield-forming characteristics of soybeans. This effect is dependent on the variety, the weather conditions, and the interaction between them. Biostimulant raw materials can be applied in combination with herbicide mixtures. The influence of these substances on chlorophyll content and the Nitrogen Index in soybean cultivation has been observed [9]. However, combining different products does not always lead to increased productivity. This was demonstrated by [10], which reported an interaction between a biostimulant and an inoculant that, in certain dosage combinations, resulted in a reduction of soybean yield.

Contemporary remote sensing technologies, including multispectral analysis, enable the precise monitoring of plant health status. These methods rely on the detection of reflected radiation across various spectral bands, allowing for the calculation of vegetation indices. This approach is grounded in the concept of precision agriculture, which involves the use of digital technologies during crop cultivation to increase yields and reduce costs while maintaining environmental stewardship. Precision agriculture employs a range of multispectral indices [11] that facilitate vegetation condition assessment, stress monitoring, and the optimization of agricultural practices. One of the most well-known indices is the Normalized Difference Vegetation Index (NDVI) [12], which is based on the ratio of reflectance in the near-infrared band to reflectance in the red band. This parameter allows for a general assessment of vegetation health and density. Other popular indices include the Soil Adjusted Vegetation Index (SAVI) [13,14] and the Normalized Difference Red Edge (NDRE) [15,16]. The selection of a specific index depends on the characteristics of the crop, environmental conditions, and the objectives of the research being conducted. In general, the use of these indices allows for a more accurate assessment of the health status of cultivated plants and facilitates the selection of precise agronomic interventions. The NDVI index can be used for soybean growth assessment [17], as well as for soybean biomass and nutrient uptake estimation [18]. A comparison of vegetation indices for detecting spatial and temporal variabilities in soybean crops using canopy sensors is presented in [19]. The NDRE index demonstrated better performance in spatiotemporal distribution mapping, while NDVI experienced saturation issues. A novel classification method for identifying high- and low-yielding soybean plots based on the NDRE index was presented in [20]. Both the NDVI and NDRE indices can be utilized to analyze the fertilization efficiency of soybean crops using nitrogen controlled-release fertilizers (CRFs) [21]. The Visible and Near-Infrared Angle Index (VNAI) can also be applied to assess chlorophyll content in soybeans [22]. Multispectral images of soybean canopies effectively reflect the physiological status and growth conditions of the plants. In [23], a multispectral image recognition method for soybean canopies based on a neural network was proposed.

The analysis of spectral indices is conducted using images of cultivated areas acquired by multispectral cameras mounted on drones or satellite systems. In the case of satellite imaging, there are significant limitations in terms of both spatial and temporal resolution. Satellite images are typically obtained every few days and offer a spatial resolution of up to 10 m. An estimation of soybean crop coverage using LANDSAT satellite data is presented in study [24]. The acquired images, with a resolution of 30 m/pixel, were the result of satellite overpasses over the analyzed area every 16 days. Satellite imaging can also be used to develop automatic soybean mapping algorithms based on combined variations in canopy water content and chlorophyll concentration [25]. Multispectral images of soybean crops can be utilized to construct drought recognition models [26]. A model using a support vector machine (SVM) achieved high accuracy and processing speed, outperforming other popular approaches.

In the case of drone-based research, it is possible to obtain imagery with a resolution reaching individual centimeters. The imaging frequency primarily depends on atmospheric conditions. However, under favorable weather, data acquisition can be carried out as frequently as desired, limited only by the availability of the drone operator. The results obtained depend on the type of camera used and the flight parameters. One significant factor affecting spectral indices is the observation angle, which is analyzed in detail in [27]. Multispectral images acquired by drone-mounted cameras can be used to delineate soybean planting areas using deep learning algorithms [28]. Moreover, machine learning techniques combined with multispectral imagery are employed for estimating soybean yield [29,30,31] and for monitoring soybean growth [32]. Soybean maturity can also be assessed using hyperspectral remote sensing approaches.

Reference [33] presents correlations between selected spectral indices and soybean maturity. The maturity dates of soybean lines can be predicted using UAV-based multispectral imagery [34]. Drone data collected with multispectral and thermal imaging sensors are also used to forecast soil moisture content during soybean cultivation [35]. As demonstrated by [36], the use of multispectral data in combination with genomic data significantly improves the accuracy of aboveground biomass prediction in soybean, particularly before flowering. Multispectral images acquired by drones, combined with machine learning algorithms, enable the classification of soybean genotypes [37]. The best results were obtained using spectral bands alone as input data. Multispectral aerial imagery can also be used to assess soybean crop germination. The authors of [38] developed software that enables automatic germination assessment, thereby accelerating the process of breeding new varieties.

High-resolution imagery from a drone equipped with a multispectral camera can be compared with lower-resolution imagery originating from satellites (e.g., Sentinel-2). Satellite-derived crop data are generated and processed within the widely used Copernicus Programme [39]. The generated images are accessible via a web browser in the Copernicus Browser [40] or EO Browser web application [41]. This approach enables the identification of crop issues using a drone, followed by the analysis of the regional context of these issues using Sentinel-2 satellite data. Utilizing the EO Browser tool, it is possible to track historical index values for a specific field from satellite data and compare them with current drone data to assess plant health and trends in their changes. Satellite platforms facilitate the comparison of the current plant status with data from previous seasons or the analysis of regional vegetation patterns. As noted in reference [42], modern unmanned aerial vehicles offer several advantages compared to conventional satellites in terms of data acquisition and analysis. Furthermore, cameras on drones can detect subtle changes in the reflectance spectrum of plants that indicate early stress (e.g., water or nutrient deficiency) before they become visible to the naked eye. This information can then be correlated with soil moisture or precipitation data from the Copernicus platform. In-field variability maps, generated from drone data, can be combined with information on soil types and historical yields, available in various information layers associated with Copernicus Programme data. Such data integration enables the calibration and validation of satellite data at a local scale. Conversely, broad-area satellite data can aid in the extrapolation of conclusions drawn from drone-based analysis to larger areas, taking into account climatic trends.

The presented approach is not solely based on a standard data compilation but creates a new quality of information, where the strength of one technology complements the weakness of the other (based on a synergy effect). An example of such an approach is the work [43], in which multispectral satellite imagery (from Formosat-2) was combined with hyperspectral imagery acquired using an unmanned aerial vehicle to improve temporal resolution in precision agriculture. A method for enhancing satellite imagery, based on deep learning techniques that utilize imagery acquired by unmanned aerial vehicles (UAVs), was presented in [44]. The potential of combining spectral information from satellite and drone cameras was also investigated in the work [45]. An analysis of the similarity of crop heterogeneity maps obtained using drones and satellites (Sentinel-2) was presented by Rasmussen et al. [46]. Other works comparing drone and satellite imagery concern the assessment of the spatial variability of yield [47], the evaluation of land surface temperature [48], and vineyard variability [49]. The combination of both types of data can also be used for growth monitoring [50] and the classification of arable land models [51].

The ultimate goal of the integration described above is to provide farmers with tools for making more precise and effective management decisions, optimizing resource consumption and increasing yields in a sustainable manner. An assessment of various data sources (satellite and UAV) related to agriculture was presented in [52].

The main research challenge addressed in this paper is the need to develop a rapid and non-invasive method for assessing soybean crop health to determine the effects of natural biostimulant application. Traditional research methods such as visual assessment, plant sampling, or soil sampling can be time-consuming or require direct intervention with plants. The effectiveness of biostimulants can depend on numerous factors, and their precise measurement is crucial from the perspective of yield evaluation and the optimization of cultivation costs. Identifying areas with potentially deteriorated crop health or areas where biostimulants should be applied is another challenge. Plant health issues are often not visible to the naked eye at early growth stages, and the use of highly sensitive measurement equipment may provide a solution to this problem. Traditional crop monitoring methods, namely visual assessment and satellite imaging, have significant limitations. In particular, the latter is characterized by low spatial resolution (typically up to 10 m or even 30 m) and temporal resolution (an image every few days) compared to crop imaging using drones. UAVs provide spatial resolution at the level of individual centimeters. The proposed approach enables the rapid and non-invasive assessment of crop health, overcoming the limitations of satellite imaging. The selection of an appropriate spectral index enables high sensitivity to changes in vegetation. For example, the NDRE index used is more sensitive to chlorophyll content in denser vegetation. This makes it useful in the later stages of plant growth, when NDVI may become saturated.

The objective of the research presented in this paper was to evaluate the feasibility of using a multispectral camera to assess the effects of natural biostimulant application in soybean cultivation. The assessment was conducted through the analysis of selected multispectral indices developed from drone-based imagery. The following indices were selected for analysis: NDRE, LCI, and OSAVI. Their detailed explanation is provided later in this paper. The values of the aforementioned indices obtained from plots treated with a natural biostimulant, a control group (without biostimulant application), and a positive control group (application of a commercial biostimulant with proven positive effects) were evaluated. The statistical analysis of selected spectral indices was conducted to detect differences in plant conditions during a specific growth stage. This allows for the evaluation of the effectiveness of the applied treatments. Following the verification of the formulated hypothesis, further research can be carried out on the selection of biopreparations and their application methods in soybean cultivation.

## 2. Methodology

### 2.1. Field Experiment

**Figure 1:**
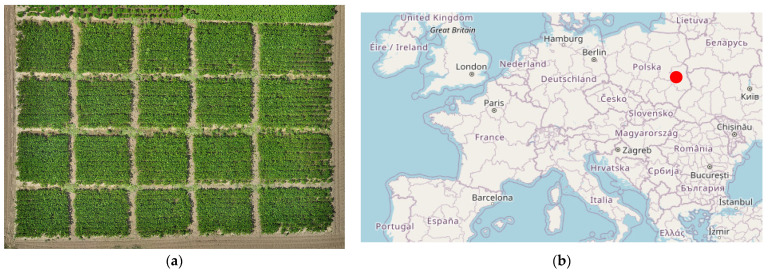
(**a**) Experimental field; (**b**) minimap of Europe with the soybean cultivation location marked (based on [53]).

The experiment was conducted in 2024 at the Czesławice Experimental Farm belonging to the University of Life Sciences in Lublin (Poland, 51.30431605 N, 22.25242636 E, 213.5 m a.s.l.). The location of the experiment is shown in Figure 1. The region where the research was carried out is characterized by a temperate continental climate. The average temperatures in June and July for this area are 22 and 23 °C, respectively, while the average rainfall is 54.0 mm and 57.6 mm.

The study utilized the Abelina soybean variety from Saatbau. It is an early (to medium-early) variety that was entered into the registry in 2016. Abelina is recommended for cultivation throughout Poland, except for regions with the most challenging thermal conditions. In the years 2014–2018, in experiments conducted by the Central Office for Plant Variety Testing in Poland (Polish: Centralny Ośrodek Badania Odmian Roślin Uprawnych, COBORU), the Abelina variety achieved record-high and stable yields, characterized by excellent early vigor and rapid canopy closure. The plant reaches approximately 103 cm in height, and its lowest pods are set high, facilitating harvesting. Abelina also stands out for its high fat and protein content. The variety is on the list of recommended varieties for cultivation in 12 provinces in Poland.

The field experiment involved the foliar application of a plant biostimulant obtained from fireweed herb in the form of an aqueous extract, the foliar application of Kelpak, and the foliar application of pure water (control combination).

Kelpak is based on an extract from the sea algae *Ecklonia maxima*, serving as a source of natural growth regulators, also known as phytohormones, primarily auxins and cytokinins. Kelpak exhibits hormonal activity, stimulating cell division, root growth, and the development of aerial organs. Furthermore, it aids in regeneration after stress, enhances flowering, and improves pod setting.

*Epilobium angustifolium* (fireweed) contains polyphenols, including flavonoids, tannins, and ellagic acid. It offers a mild stimulating effect, bolsters plants’ natural resistance to pathogens, and increases their antioxidant potential.

Kelpak is a ready-to-use commercial product with good chemical stability. In contrast, a biostimulant based on *Epilobium angustifolium* is prepared independently (via maceration or infusion in water). It has a short shelf life, and its application involves standardization challenges due to variable concentrations of active substances. There are a limited number of studies on its use as a biostimulant for cultivated crops. This plant extract potentially improves overall plant vitality, though its effects might be less pronounced and more long-term. Given this potential, it could be utilized in areas such as organic farming, necessitating further research into its effectiveness in crop cultivation.

The fireweed herb extract was prepared as an infusion in three variants differing in the herb-to-extractant ratio (25 g/1000 mL; 50 g/1000 mL; 75 g/1000 mL). These biostimulants were prepared by extracting dried biomass from the aerial parts of *Epilobium angustifolium* in 1000 mL of water for 30 min at 95 °C. The extracts obtained in this way were centrifuged at 4250 rpm for 5 min. The Kelpak biostimulant was used in the experiment at a concentration of 1.0%. The control combination was sprayed with pure water in the same amount as the biostimulant combinations.

The *E. angustifolium* extracts were applied to investigate their potential as a biostimulant for leguminous plants. The Kelpak biostimulant was used as a positive control to validate the produced plant extracts. Previous studies on Kelpak in soybean cultivation have proven its positive effects on plant biometric characteristics and seed yield and quality [4,54].

Soybean seeds of the Abelina variety were sown with a precision planter on 6 May. A row spacing of 33.3 cm and a seed spacing of 4.0 cm within the row were used, resulting in a planting density of 75,000 seeds per hectare.

**Figure 2:**
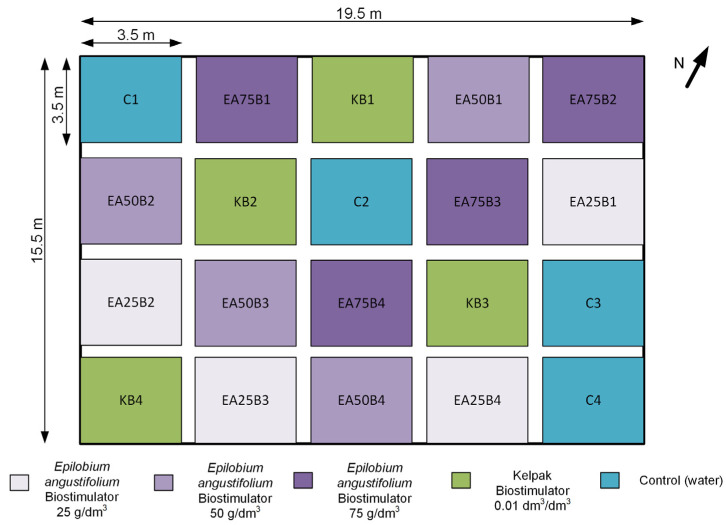
Design of the field experiment.

Within the area designated for soybean cultivation, experimental plots with dimensions of at least 3.5 × 3.5 m each were marked out (Figure 2). The entire area consisted of twenty plots, which were randomly divided into five categories: EA25, EA50, EA75—soybean plants treated with the produced plant biostimulant at three concentrations; KB—positive control combination: soybean plants treated with Kelpak biostimulant; C—control combination: soybean plants treated with water. Each category was replicated four times across the designated area, while the last digit in the plot code was introduced to distinguish them. The adopted concentration values were based on the authors’ previous experiences in the field of crop biostimulation. Soybean seeds were sown with a precision planter in the first ten days of May.

On 19 June 2024, the plant biostimulant in the form of fireweed extract, the Kelpak biostimulant (positive control), and the control (sprayed with pure water) were applied to the experimental field. The working liquid dose was 250 L/ha.

To verify the homogeneity of soil conditions, soil tests were conducted at three test locations. The soil was classified as Haplic Luvisol (silt loam). The first three measurements were carried out before biostimulant application, and the subsequent three after application. Soil samples were taken as averages from plots of each combination, specifically, (1) plots where the extract would be applied, (2) plots where Kelpak would be applied, (3) plots with water control, (4) plots after water application and soybean harvest, (5) plots after Kelpak application and soybean harvest, and (6) plots after extract application and soybean harvest.

The pH parameter had a value of 6.1 before application and 6.7 after application. The content of available forms of mineral nutrients in the soil, including phosphorus, potassium, magnesium, boron, manganese, copper, zinc, and iron, was also analyzed. The average phosphorus content before biostimulant application was 25.4 mg/100 g of soil, potassium content was 14.7 mg/100 g of soil, and magnesium content was 6.9 mg/100 g of soil. After application, these values were 32.0 mg/100 mg of soil, 17.6 mg/100 g of soil, and 5.2 mg/100 g of soil, respectively. Detailed results, including the content of other mineral nutrients, are presented in Table 1 and Table 2. The results of the soil tests before application indicate the homogeneity of soil conditions for each tested combination.

Initially, the soil in all variants exhibited very similar physicochemical parameters. The pH was slightly acidic (6.1), indicating favorable growing conditions for soybeans. Phosphorus and potassium content was moderate, while magnesium content was very good. Micronutrients were present in sufficient quantities, suggesting no deficiencies. This indicates that initial conditions were balanced and comparable, lending credibility to subsequent observations.

Significant changes in the soil’s chemical composition were observed after soybean harvest. An increase in pH to approximately 6.7–6.8 was noted in every plot. Specifically, plots treated with biostimulants showed higher levels of phosphorus and potassium compared to the water control. The plot treated with the *Epilobium angustifolium* extract exhibited the largest increase in phosphorus content (by 9.4 mg/0.1 kg soil) and a relatively smaller decrease in magnesium and copper, which may suggest an improved soil mineral economy. Additionally, the observed increase in manganese content and maintenance of zinc levels in biostimulant-treated plots indicate a beneficial effect on microbiological activity and trace element stability.

The collected data suggest that the application of natural biostimulants, in the form of both sea algae and plant extracts, can lead to beneficial changes in the soil environment, fostering improved nutrient uptake efficiency by plants. These results form the basis for further correlation analyses between soil parameters, multispectral signals, and soybean yield efficiency.

At the end of the vegetation period (before seed harvest), the following biometric measurements were taken: plant height, first pod height, and number of pods per plant.

### 2.2. Drone with Multispectral Camera

**Figure 3:**
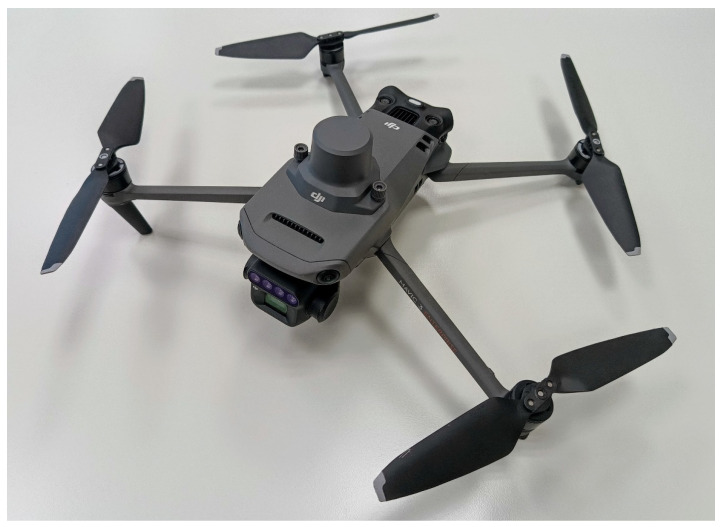
The drone with a multispectral camera used for the research.

The research was conducted using a DJI Mavic 3 Multispectral (SZ DJI Technology Co., Ltd., Shenzhen, Guangdong, China), a lightweight and compact drone designed for ease of use and mobility (Figure 3). Its foldable design facilitates transportation and storage, which is particularly important for fieldwork. The drone is equipped with an advanced navigation system that enables precise positioning and stable flight, even in challenging atmospheric conditions. The Mavic 3 Multispectral offers a range of intelligent features that simplify task execution, such as automatic take-off and landing, flight path planning, and object tracking. The drone also incorporates an obstacle avoidance system, which enhances flight safety and minimizes the risk of collisions. The drone’s flight time is up to 43 min, allowing for the coverage of areas up to 200 hectares in a single flight. The drone is compatible with various applications that enable flight planning, data analysis, and report generation.

The Mavic 3 Multispectral utilizes a GNSS (Global Navigation Satellite System) for accurate spatial positioning. Additionally, it can be equipped with an RTK (Real-Time Kinematic) module, which significantly improves positioning accuracy by utilizing data from reference stations. This allows for an increase in accuracy to below 2 cm.

**Figure 4:**
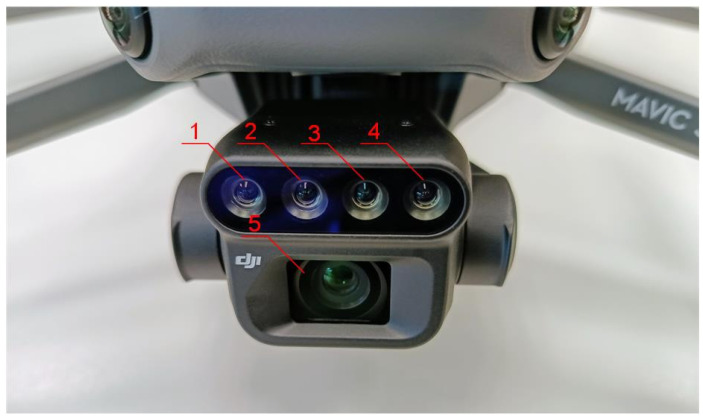
Cameras used for the research: 1—near-infrared lens; 2—red edge band lens; 3—red band lens; 4—green band lens; 5—RGB lens.

The multispectral camera (Figure 4) in the DJI Mavic 3 Multispectral drone comprises four lenses, each capturing light in a different spectral range: green, red, red edge, and near-infrared. Additionally, the drone is equipped with a 20 MPx RGB camera, enabling the acquisition of images in the standard visible spectrum. Multispectral cameras are widely used for crop health analysis. They enable the estimation of yields [55], the detection of water stress [56] and disease [57], and the monitoring of leaf nutrient content [58]. There are several models of such cameras available on the market, differing primarily in matrix resolution, the number and range of recorded bands (spectral resolution), and radiometric resolution. The camera model selected for the research is directly integrated with the drone by the manufacturer. This integration also extends to the mission planning software, forming a compatible system. Other devices of this type available on the market (e.g., from MicaSense or Sentera) are installed on the drone as external equipment. The selected technical parameters of the camera used for the research are presented in Table 3.

The drone flight parameters were planned to ensure proper coverage of the area, which was less than 700 m^2^. The drone captured 23 images, traversing a 96 m-long route at an average speed of 2.2 m/s. The operation lasted 47 s, during which the aircraft maintained an altitude of 25 m relative to the take-off point, capturing images in Timed Interval Shot mode with 70% side overlap and 80% front overlap. The adopted settings allowed for a ground sampling distance (GSD) of 1.16 cm/pixel. A summary of the key mission parameters is presented in Table 4.

**Figure 5:**
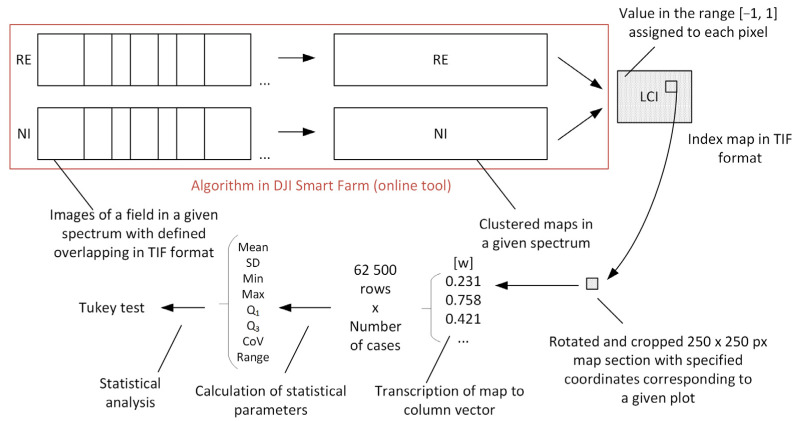
Diagram illustrating the post-processing of the recorded images.

The conducted research involved performing a scan of a field experiment where Abelina variety soybeans treated with biostimulants were cultivated. The imaging of the experiment was carried out using a drone-mounted multispectral camera. The post-processing of the recorded images enabled the quantitative and qualitative analysis of the generated data. A diagram illustrating the applied data processing procedure is shown in Figure 5. The obtained index maps underwent mathematical processing. Fragments with dimensions of 250 × 250 pixels, corresponding to a specific experimental plot, were extracted from the full field image. Subsequently, the extracted map fragment was converted into a column vector containing the values of the given multispectral index within the range of [−1; 1]. Following this, statistical parameters in the form of the mean value and standard deviation were calculated for each plot.

### 2.3. Field Experiment Scanning

**Figure 6:**
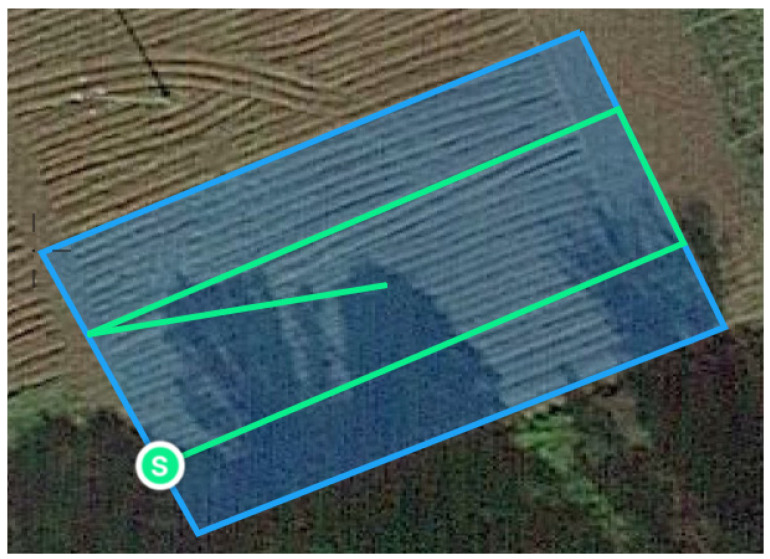
Drone flight trajectory during the mission.

Figure 6 shows the drone’s trajectory, automatically determined by the autopilot algorithm based on the input mapping area. The diamond symbol indicates the take-off and landing location, while the *S* symbol indicates the point where aerial photography of the area begins.

Good lighting conditions prevailed during the flights. The flights were conducted during sunny weather, without rainfall or strong winds. A detailed flight schedule with corresponding weather conditions (air temperature and humidity) is presented in Table 5. The application was performed at the BBCH 13–14 growth stage, while observation was conducted at the BBCH 61 stage.

**Figure 7:**
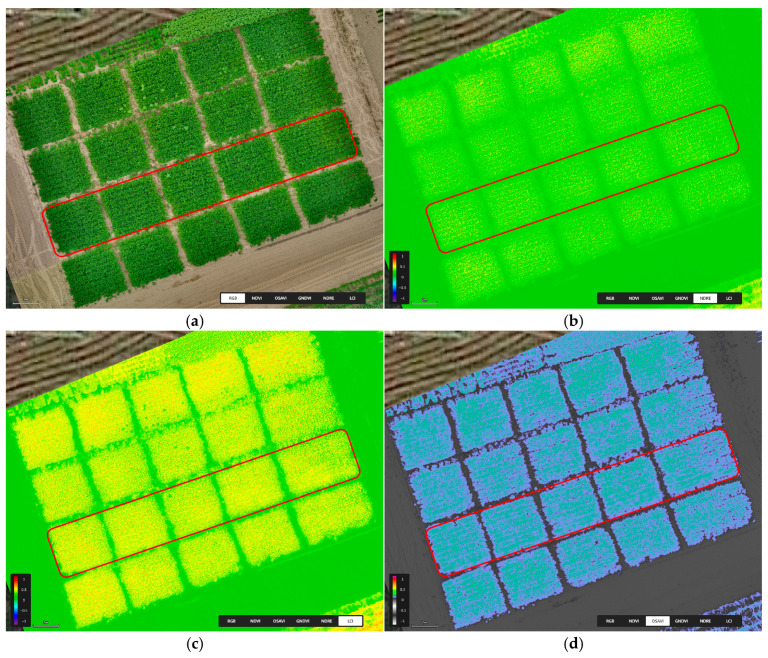
(**a**) The obtained RGB image; (**b**) NDRE; (**c**) LCI; (**d**) OASVI spectral maps one day before the application of biostimulants.

The spectral maps presented in Figure 7 were developed using the DJI Smart Farm Web tool [59]. Detailed parameter analysis was performed using GNU Octave 9.4.0 (GNU General Public License, GNU Project, Software Foundation, Boston, MA, USA) [60], Microsoft Excel 2019 (Microsoft Corporation, Redmond, Washington, DC, USA) and Statistica 13.3 (TIBACO, San Ramon, CA, USA). For the experimental plots marked with a red line, the index values were calculated, and the results were statistically compared 20 days after the application.

### 2.4. Vegetation Indices

The first analyzed index was the NDRE (Normalized Difference Red Edge Index). This parameter is similar to the NDVI (Normalized Difference Vegetation Index), but it is more sensitive to chlorophyll content in denser vegetation. This allows for the early detection of stresses related to nutrient deficiencies or unfavorable environmental conditions. Its significance is greater in the later stages of plant growth, when the NDVI is no longer sufficiently sensitive to changes occurring in the plant biomass. This parameter takes into account reflectance in the near-infrared and red edge bands (Equation (1)).

(1) $$NDRE=\frac{NIR-RE}{NIR+RE}$$

where

*NIR*—reflectance in the near-infrared range;

*RE*—reflectance in the red edge range.

The NIR and RE values appearing in the formula represent the reflected portion of incident radiation. The spectral camera captures light reflected from the surface in the form of multispectral images. In contrast, the sunlight sensor records the light incident on the analyzed surface.

The LCI (Leaf Chlorophyll Index) is determined using a similar mathematical relationship (Equation (2)) as the NDRE index. The difference lies in the wavelengths that are taken into account for calculating the final index value. This index provides more precise information about chlorophyll content compared to the NDRE index.

(2) $$LCI=\frac{NIR-RE}{NIR+G}$$

where

*NIR*—reflectance in the near-infrared range;

*RE*—reflectance in the red edge range;

*G*—reflectance in the green range.

The last of the considered indices is the OSAVI (Optimized Soil-Adjusted Vegetation Index). This parameter takes into account the type of substrate during the analysis of the vegetation condition. The index allows for image correction in situations where areas of bare soil are visible between the studied vegetation. The OSAVI parameter reduces the influence of visible soil on the analysis result and is effective in areas with low vegetation cover. The correction is performed using the calibration constant presented in Equation (3).

(3) $$OSAVI=\frac{\left(NIR-R\right)}{NIR+R+C}$$

where

*NIR*—reflectance in the near-infrared range;

*R*—reflectance in the red range;

*C* = 0.16—a calibration constant that minimizes the influence of soil.

The analysis utilized results obtained from scanning the following plots: EA25B2, EA25B3, EA75B4, KB3, C3. The obtained mapping results were processed and subjected to statistical analysis aimed at evaluating the suitability of multispectral camera scanning for assessing the effects of applying the produced plant biostimulant in the form of fireweed extract, a positive control combination using Kelpak, and a water control in soybean cultivation.

### 2.5. Statistical Analysis

The data obtained from multispectral scanning and biometric measurements of plants were subjected to statistical analysis by calculating the values of means, standard deviations, minimum value, maximum value, and range. To examine the occurrence of significance of differences between the studied variables, the Tukey test was used at the significance level of *p* < 0.05. Before the Tukey test was used, the normality of the distribution of variables and the homogeneity of variance were checked. The calculated vegetation indices were correlated with the results of biometric measurements.

## 3. Results and Discussion

The characteristics of the obtained values for the analyzed indices for the tested applications are presented in Table 6. The lowest minimum NDRE index value was obtained in the plot where the *E. angustifolium* extract prepared at a ratio of 50 g of herb per 1000 mL of water was applied, and in the control combination where pure water was used. The highest values for this index were recorded for the control. Although the largest range was observed in the control combination, the greatest variability, determined by the coefficient of variation, was observed for the combination where a 1% Kelpak solution was applied. Similarly, for the LCI and OSAVI indices, the coefficient of variation had the highest value for plants treated with Kelpak. The smallest range values for the LCI index occurred in combinations treated with *E. angustifolium* extracts prepared at ratios of 25 and 50 g per 1000 mL of water.

**Figure 8:**
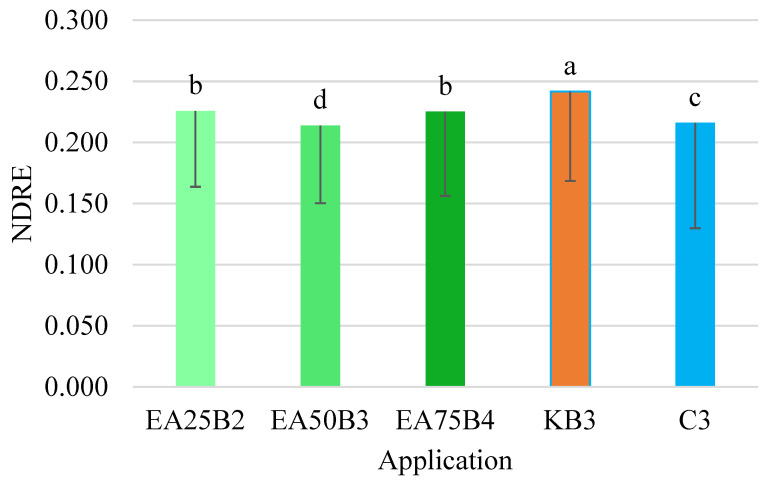
NDRE index values for the tested preparation applications (mean and *SD*). Values marked with different letters differ significantly at *p* < 0.05.

Statistical analysis revealed significant differences between the values of individual indices and the applied fireweed preparation compared to the controls. As expected, the value of the chlorophyll content analysis-based index (NDRE) was highest after the application of the Kelpak biostimulant and differed significantly from the other combinations (Figure 8). The control combination had almost the lowest value for this index. The suitability of the NDRE index for assessing soybean productivity was evaluated by Miller et al. [20] using a RapidSCAN CS-45 Handheld Crop Scanner (Holland Scientific, Lincoln, NE, USA). The authors of this study demonstrated the usefulness of this index for assessing soybean productivity at various growth stages.

**Figure 9:**
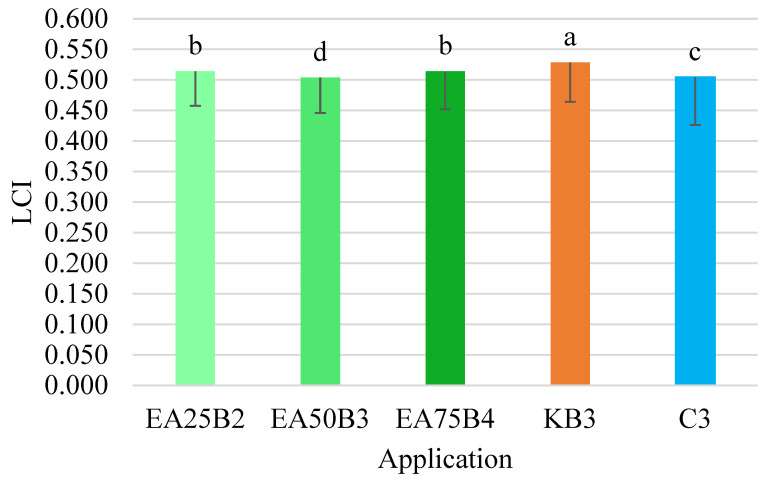
LCI index values for the tested preparation applications (mean and *SD*). Values marked with different letters differ significantly at *p* < 0.05.

The LCI index values for the tested combinations ranged from 0.504 to 0.529. The highest value for this index, as with the NDRE index, was obtained for the combination where the Kelpak biostimulant was applied (Figure 9). This value differed significantly from the values obtained in the other combinations. Similar to the NDRE index, the lowest average LCI index value was obtained in the combination where the *E. angustifolium* extract was applied at a ratio of 50 g per 1000 mL of water (EA50B3).

**Figure 10:**
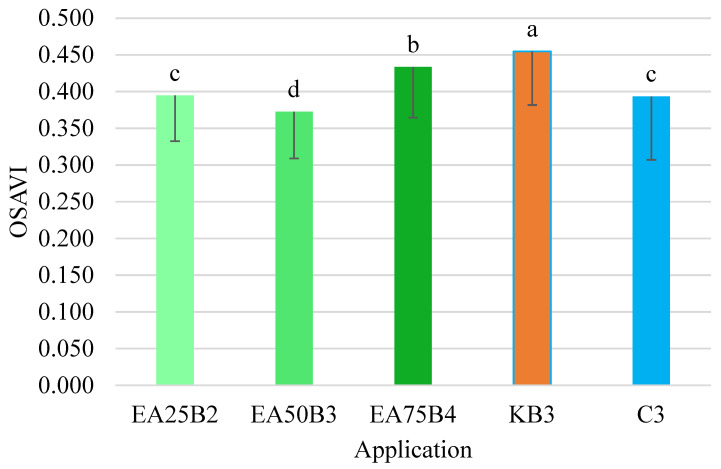
OSAVI index values for the tested preparation applications (mean and *SD*). Values marked with different letters differ significantly at *p* < 0.05.

The OSAVI index values were the most varied among the tested combinations (Figure 10). Despite this variability, the obtained values confirmed the relationships demonstrated during the analysis of the NDRE and LCI indices. It was found that the OSAVI index value for the combination where Kelpak was applied was the highest and differed significantly from the values of this index for the individual combinations. The suitability of the OSAVI index in studies on leaf area index (LAI) was investigated by Liu et al. [61]. They demonstrated a high correlation between the studied OSAVI index and LAI, as evidenced by the coefficient of determination R^2^ = 0.83.

It was observed that for all analyzed indices, the second highest value was obtained after the application of the *E. angustifolium* extract prepared at a ratio of 75 g per 1000 mL of water. Research by Voitik et al. [62] also indicates the potential of using multispectral cameras in assessing the condition of crops such as barley, corn, and soybeans, especially under abiotic stress conditions such as soil salinity.

The tallest plants were observed following the application of the E. angustifolium biostimulant prepared at a ratio of 25 g of herb per 1000 mL of water (EA25B2) (Table 7) to soybean plants. These plants differed significantly in height from those treated with the E. angustifolium biostimulant prepared at a ratio of 50 g of herb per 1000 mL of water (EA50B3) and the control group where no biostimulants were applied (C3). The height of the first pod was similar across almost all studied variants. Only plants treated with the biostimulant from E. angustifolium prepared at a proportion of 75 g of herb per 1000 mL of water (EA75B4) exhibited the smallest first pod height, which was significantly different from the remaining combinations. In the combinations where biostimulants were used, the number of pods per plant did not differ significantly. The lowest number of pods per plant was found in the control variant (no biostimulant application), and this differed significantly from all other variants.

The calculated Pearson correlation coefficients between the vegetation indices LCI, NDRE, and OSAVI and plant height indicate a strong positive relationship for LCI (*R* = 0.66) and NDRE (*R* = 0.64), and a moderate positive relationship for OSAVI (*R* = 0.43). The correlation coefficients between the vegetation indices and the first pod height showed a weak negative correlation for LCI (*R* = −0.23) and NDRE (*R* = −0.21), and a strong negative correlation for OSAVI (*R* = −0.61). Conversely, a strong positive correlation was found between the LCI index and the number of pods per plant (*R* = 0.63). Moderate positive correlations were observed between the NDRE and OSAVI indices and the number of pods per plant (*R* = 0.58 and 0.57, respectively).

Lee et al. [63] analyzed the correlation coefficients of the OSAVI and NDRE indices with dry matter hay yield, demonstrating a positive correlation for both indices. These findings confirm the utility of multispectral scanning for assessing crop yield. However, the authors noted that the variability of plant material across different sampling locations influenced the accuracy of biomass estimates obtained from remote sensing data. Poor clarity and resolution of images in multispectral data from unmanned aerial vehicles (UAVs) affect the accuracy of regression models used for biomass estimation. The utility of multispectral scanning using UAVs for monitoring corn cultivation under various irrigation conditions was demonstrated by Ma et al. [64]. Strong correlations were found between the calculated LCI and NDRE indices and leaf pigment content. The need for further research to confirm the observed relationships was highlighted.

Using a drone equipped with a multispectral camera provides an effective tool for assessing plant status. Compared to manual multispectral scanners (e.g., PolyPen RP410, CID Bio-Science CI-710), the drone-based method allows for rapid data acquisition from large experimental areas. This method simultaneously reduces operator influence and eliminates errors associated with random sampling. Although manual scanners offer high accuracy at the single-leaf level, they are time-consuming and labor-intensive for large-scale crop observations. Chemical analyses of collected plant parts (e.g., macro- and micronutrient content, dry matter, chlorophyll) offer very high precision but require sample collection and processing in specialized analyzers. These methods are time-consuming, costly, and inefficient for dynamic crop monitoring during vegetation. Furthermore, results are only available after the analysis is completed, which limits the ability to make rapid agrotechnical decisions. Chlorophyll meters (e.g., SPAD-502Plus) enable the quick and non-destructive assessment of chlorophyll content in leaves. However, this measurement is point-based, which limits its representativeness, especially in cases of high variability within a crop. Results also depend on leaf age and thickness, its position on the plant, and its angle of inclination, making data comparability difficult.

In contrast to the above methods, analyzing vegetation indices obtained from a drone allows for a comprehensive assessment of crop health. The resulting maps can be analyzed quantitatively, spatially, and temporally. Positive correlations between these indices and phenotypic traits (such as plant height or pod count) confirm this technology’s potential for evaluating the effectiveness of biostimulants in crop cultivation. While the aforementioned traditional methods remain useful for validation and basic research, the method utilizing a drone with a multispectral camera offers a non-invasive, scalable, and rapid way to monitor large crop areas and their responses to various cultivation practices.

The research presented in this study also demonstrates strong correlations between the LCI and NDRE indices. However, considering the existing limitations in the presented research (the small area of studied plots, a single soybean variety, biometric measurements taken only at the end of the field experiment, and the absence of stress factors during plant vegetation), future works should focus on a more precise investigation of the correlations between indices obtained through multispectral scanning and biometric measurement results. To achieve this, experiments should be conducted on larger cultivation areas, incorporating different soybean varieties and performing biometric measurements directly after each multispectral scan. This will allow for a more accurate assessment of the utility of vegetation indices such as LCI, NDRE, and OSAVI for the rapid and precise evaluation of soybean plant health.

## 4. Conclusions

The utilization of drone-mounted multispectral cameras creates new research opportunities in the context of crop analysis. The conducted analysis of NDRE, LCI, and OSAVI index values demonstrated the potential for monitoring crop status after the application of both commercial biostimulants, such as Kelpak, and those produced in the form of aqueous extracts from medicinal plants. The highest NDRE index values were observed after the application of the Kelpak biostimulant, and this value differed significantly from the other combinations. The control combination (pure water) showed almost the lowest value for this index. Similarly to NDRE, the highest LCI index value was obtained for the combination with the Kelpak biostimulant, and this value also differed significantly from the others. The lowest average LCI index value was recorded in the combination with the *E. angustifolium* extract at a concentration of 50 g per 1000 mL of water. The OSAVI index value for the Kelpak combination was the highest and differed significantly from the values for the other combinations. For all analyzed indices (NDRE, LCI, OSAVI), the second highest value was obtained after the application of the *E. angustifolium* extract at a ratio of 75 g per 1000 mL of water. The greatest variability (determined by the coefficient of variation) for the NDRE, LCI, and OSAVI indices was observed in the combination where a 1% Kelpak solution was applied. The smallest range of values for the LCI index occurred in combinations treated with *E. angustifolium* extracts at concentrations of 25 and 50 g per 1000 mL of water. Statistical analysis confirmed the existence of significant differences between the values of individual indices and the applied fireweed preparation compared to the controls.

Kelpak-treated plots exhibited the greatest spectral variability, particularly in the NDRE, LCI, and OSAVI indices, suggesting heterogeneous plant responses, potentially due to microenvironmental factors or foliar uptake variability. In contrast, fireweed extract treatments at lower concentrations (25–50 g∙L^−1^) yielded more uniform spectral responses.

Correlation analysis revealed strong positive relationships between LCI and plant height and between NDRE and plant height, indicating that both indices effectively reflect vertical growth dynamics. A strong negative correlation between OSAVI and the first pod height suggests a potential inverse relationship between lower pod set and spectral density, while the number of pods per plant showed the highest correlation with LCI, confirming the potential of this index as an indicator of yield components.

The application of natural biostimulants—Kelpak (seaweed-based) and a willowherb (*Epilobium angustifolium*) aqueous extract—induced measurable changes in soil chemical properties in a soybean cultivation system. Despite similar initial soil conditions across plots, post-harvest analyses revealed increased levels of phosphorus and potassium in biostimulant-treated soils, with the willowherb extract showing the most pronounced effect on phosphorus accumulation. Additionally, the extract-treated plots exhibited a more favorable retention of magnesium and copper compared to the control. Elevated manganese and stable zinc levels suggest enhanced micronutrient dynamics, potentially linked to improved microbial activity. These findings indicate that natural biostimulants may influence plant nutrition and provide a foundation for further multispectral correlation studies. Future studies could ensure more homogeneous initial soil conditions or apply statistical correction models (e.g., ANCOVA or multivariate regression) to accurately isolate the effect of biostimulants on crop performance.

The results demonstrate that the vegetation indices LCI and NDRE are strongly correlated with plant height, suggesting their usefulness in estimating crop biomass. OSAVI showed a moderate correlation with plant height but exhibited a strong negative correlation with the height of the first pod, indicating its potential for assessing lower canopy development. Additionally, all three indices showed positive correlations with the number of pods per plant, with LCI being the most strongly correlated. These findings highlight the potential of multispectral vegetation indices, particularly LCI and NDRE, for monitoring key agronomic traits in crops.

The finding of significant differences based on the values of the aforementioned indices demonstrated the capability of the proposed non-invasive method for diagnosing the status of soybean crops. However, to fully exploit the potential of multispectral cameras for evaluating the effects of biostimulant application, it is necessary to expand the range of analyzed indices and correlate them with the biological effects of applying these preparations.

## Figures and Tables

**Table 1:** Soil pH and mineral content.

Measurement Point	pH	mg/0.1 kg Soil		
		Phosphorus	Potassium	Magnesium
1	6.1	24.0	14.5	6.5
2	6.1	25.9	14.5	7.2
3	6.1	26.3	15.1	7.0
4	6.7	30.2	16.8	4.9
5	6.7	32.5	19.0	4.9
6	6.8	33.4	17.0	5.7

**Table 2:** Soil mineral content (continued).

Measurement Point	mg/1 kg Soil				
	Boron	Manganese	Copper	Zinc	Iron
1	0.76	206.0	3.3	12.8	1168
2	0.80	169.0	3.1	12.5	1154
3	0.79	183.0	3.1	12.5	1214
4	0.99	239.0	2.6	7.3	1272
5	0.95	248.0	2.8	8.3	1223
6	0.76	234.0	2.9	8.5	1248

**Table 3:** Parameters of the cameras used for the research.

Parameter	Value/Description
RGB camera	
Resolution	20 MPx (5280 × 3956 px)
Field of view	84°
Equivalent focal length	24 mm
Aperture	f/2.8 to f/11
Shutter speed	Electronic shutter: 8–1/8000 s
Multispectral camera	
Resolution	5 MPx (2592 × 1944 px)
Field of view	73.91°
Equivalent focal length	25 mm
Aperture	f/2.0
Shutter speed	Electronic Shutter: 1/30–1/12,800 s
Central wavelength of the green band ^1^	560 nm (32 nm)
Central wavelength of the red band ^1^	650 nm (32 nm)
Central wavelength of the red edge band ^1^	730 nm (32 nm)
Central wavelength of the near infrared band ^1^	860 nm (52 nm)

^1^ Bandwidth is given in parentheses.

**Table 4:** Parameters of the flight mission.

Parameter	Value/Description
Mapping area	669.7 m^2^
GSD	1.16 cm/pixel
Distance	96.0 m
Estimated duration	47 s
Photos	23
Mode	Ortho Collection
Altitude mode	Relative to Takeoff Point (ALT)
Route altitude	25.0 m
Speed	2.2 m/s
Course angle	67°
Side overlap ratio	70%
Frontal overlap ratio	80%
Margin	0 m
Photo mode	Timed Interval Shot

**Table 5:** Schedule of flights performed over the experimental crop and corresponding atmospheric conditions.

Research Phase	Date	Days Before/ After Application	Air Temperature	Relative Air Humidity
Before application	18 June	1	27 °C	50%
Application	19 June ^1^	0	28 °C	45%
After application	9 July	20	28 °C	46%

^1^ No flight was conducted on this date.

**Table 6:** Characteristics of the NDRE, LCI, and OSAVI index values obtained for the plots 20 days after biostimulant application.

Application Code	Min	Max	1st Quartile	3rd Quartile	Coefficient of Variation	Range
NDRE (Normalized Difference Red Edge Index)						
EA25B2	−0.035	0.498	0.184	0.263	27.5%	0.533
EA50B3	−0.106	0.561	0.176	0.255	29.7%	0.667
EA75B4	−0.255	0.600	0.176	0.271	30.6%	0.855
KB3	−0.192	0.616	0.161	0.271	39.9%	0.596
C3	−0.129	0.639	0.192	0.286	30.3%	0.769
LCI (Leaf Chlorophyll Index)						
EA25B2	0.255	0.733	0.475	0.553	11.0%	0.478
EA50B3	0.169	0.765	0.467	0.537	11.5%	0.596
EA75B4	−0.035	0.796	0.475	0.553	12.1%	0.831
KB3	0.059	0.804	0.459	0.553	15.5%	0.745
C3	0.145	0.820	0.482	0.569	12.3%	0.675
OSAVI (Optimized Soil-Adjusted Vegetation Index)						
EA25B2	−0.302	0.820	0.318	0.482	31.9%	1.122
EA50B3	−0.318	0.835	0.302	0.459	34.1%	1.153
EA75B4	−0.255	0.827	0.365	0.522	29.2%	1.082
KB3	−0.318	0.859	0.318	0.490	37.4%	1.176
C3	−0.263	0.937	0.380	0.537	28.4%	1.200

EA25B2—*E. angustifolium* extract (25 g herb per 1000 mL), EA50B3—*E. angustifolium* extract (50 g herb per 1000 mL), EA75B4—*E. angustifolium* extract (75 g herb per 1000 mL), EAKB3—1% Kelpak solution, C3—pure water.

**Table 7:** Biometric measurement results (average ± standard deviation).

Application Code	Plant Height (cm)	First Pod Height (cm)	Number of Pods per Plant
EA25B2	125.2 ± 1.7 a	14.9 ± 2.8 a	29.8 ± 4.1 a
EA50B3	113.7 ± 6.7 bc	13.0 ± 0.9 ab	29.8 ± 6.9 a
EA75B4	120.1 ± 3.9 ab	10.1 ± 1.1 b	35.3 ± 1.0 a
KB3	121.0 ± 4.9 ab	11.8 ± 0.8 ab	33.8 ± 1.6 a
C3	106.2 ± 1.3 c	12.4 ± 1.5 ab	18.8 ± 2.3 b

EA25B2—*E. angustifolium* extract (25 g herb per 1000 mL), EA50B3—*E. angustifolium* extract (50 g herb per 1000 mL), EA75B4—*E. angustifolium* extract (75 g herb per 1000 mL), EAKB3—1% Kelpak solution, C3—pure water. Values in columns marked with different letters differ significantly at *p* < 0.05.

## Data Availability

Data are available on request due to privacy restrictions.
